# When a Feller Is Off’n His Feed

**Published:** 1899-06

**Authors:** 


### When a Feller is Off'n His Feed.

---

All Nature is bilious from her heels to her head
When a feller is off'n his feed.
She's all out of humor, she's in deepest despair
When a feller is off'n his feed.
There ar'nt no joy in the earth, no good in the sea;
Ain't no "ginger" in life as there on'ct used to be
When a feller is off'n his feed.
W'ats th' good of blue skies an' blossomin' trees
When a feller is off'n his feed?

It makes little difference 'bout the patch on his knees
When a feller is off'n his feed.
All he can see are the clouds, which look so big to his eye
They shet out the landscape and kiver the sky,
An' the sun can't shine through 'em th' best it can try
When a feller is off'n his feed.

Y'er ain't worth a cuss for th' work of this earth
When a feller is off'n his feed.
Y'er feels the whole blund'ring mistake of yer birth
When a feller is off'n his feed.
Y'er feels you've no int'rest in th' whul fool plan,
Sorter been slighted by Natur's cute han',
A dejected, left over, disconsolat' man
When a feller is off'n his feed.

Y'er have lost your stiff grip on the whul of th' crowd
When a feller is off'n his feed.
Feel like a dead man with nary a shroud
When a feller is off'n his feed.
You may be crawlin' aroun' but your out of the game;
Tediously groan and grunt but y'er dead jus' th' same,
Y'er dead, and ther's no preacher to boost yer to fame—
When a feller is off'n his feed.—*Ex.*

